# Recurrent Non-Variceal Upper Gastrointestinal Bleeding among Patients Receiving Dual Antiplatelet Therapy

**DOI:** 10.3390/diagnostics13223444

**Published:** 2023-11-14

**Authors:** Ah Young Yoo, Moon Kyung Joo, Jong-Jae Park, Beom Jae Lee, Seung Han Kim, Won Shik Kim, Hoon Jai Chun

**Affiliations:** 1 Division of Gastroenterology, Department of Internal Medicine, Korea University Guro Hospital, Korea University College of Medicine, 148, Gurodong-ro, Guro-gu, Seoul 08308, Republic of Korea; person88@naver.com (A.Y.Y.); gi7pjj@korea.ac.kr (J.-J.P.); l85210@korea.ac.kr (B.J.L.); kimseunghan09@gmail.com (S.H.K.); ws907568@gmail.com (W.S.K.); 2 Division of Gastroenterology, Department of Internal Medicine, Korea University Anam Hospital, Korea University College of Medicine, 73, Inchon-ro, Seongbuk-gu, Seoul 02841, Republic of Korea; drchunhj@gmail.com

**Keywords:** dual antiplatelet therapy, upper gastrointestinal tract, bleeding, mortality

## Abstract

Background: Patients undergoing dual antiplatelet therapy (DAPT) may experience recurrent gastrointestinal bleeding (GIB). We investigated the clinical characteristics and risk factors for recurrent non-variceal upper gastrointestinal bleeding (NVUGIB) in patients who had experienced NVUGIB while receiving DAPT. Methods: We enrolled patients diagnosed with NVUGIB while receiving DAPT between 2006 and 2020. Definite bleeding was confirmed by esophagogastroduodenoscopy in all NVUGIB patients. Results: A total of 124 patients were diagnosed with NVUGIB while receiving DAPT. They were predominantly male (*n* = 103, 83.1%), bleeding mostly from the stomach (*n* = 94, 75.8%) and had peptic ulcers (*n* = 72, 58.1%). After the successful hemostasis of NVUGIB, 36 patients (29.0%) experienced at least one episode of recurrent upper GIB, 19 patients (15.3%) died, and 7 (5.6%) patients had a bleeding-related death. Multivariate analysis showed that age was a significant factor for re-bleeding (odds ratio [OR], 1.050; 95% confidence interval [CI]: 1.001–1.102; *p*-value: 0.047), all-cause mortality (OR, 1.096; 95% CI: 1.020–1.178, *p* = 0.013), and re-bleeding-related mortality (OR, 1.187; 95% CI: 1.032–1.364, *p*-value: 0.016). In Kaplan–Meier analysis, the cumulative probabilities of re-bleeding, death, and bleeding-related death were significantly higher in patients aged 70 and older (*p* = 0.008, <0.001, and 0.009, respectively). Conclusions: Clinicians should be cautious about re-bleeding and mortality in elderly patients who experience NVUGIB while receiving DAPT.

## 1. Introduction

Hemorrhage of the gastrointestinal (GI) tract is a major disease; more than 500,000 patients are hospitalized in the United States, and the readmission rate is the highest. Bleeding from the upper GI (UGI) tract, including the esophagus, stomach, and duodenum, accounts for approximately 40% of the bleeding from the entire GI tract [1]. Apart from hemorrhage of the esophageal or gastric varices, non-variceal UGI bleeding (NVUGIB) continues to increase owing to the aging population and the increased use of nonsteroidal anti-inflammatory drugs (NSAIDs) and antiplatelet agents [2]. Antiplatelet agents, such as low-dose aspirin (LDA) or the more potent clopidogrel, P2Y12 inhibitors, cilostazol, and sarpogrelate, are commonly used drugs that are effective in the prevention of acute coronary syndrome (ACS), peripheral vascular disease, and cerebrovascular accident (CVA) [3]. Dual antiplatelet therapy (DAPT) is the mainstay of management of acute vascular events and maintenance therapy after percutaneous intervention [4]. However, the long-term use of antiplatelet agents or DAPT is significantly associated with major hemorrhage in the GI tract, which may lead to re-hospitalization or mortality after acute vascular events [5].

Previous studies have shown that the incidence of 30-day NVUGIB among patients who underwent percutaneous coronary intervention (PCI) for ACS was 1.2–1.3% [6,7], and a recent nationwide study in the US showed that the 11-month incidence of NVUGIB after PCI for the management of acute myocardial infarction was 1.6%, and 11-month mortality was significantly associated with the development of NVUGIB [8]. Several clinical features, such as old age, anemia, thrombocytopenia, renal insufficiency, malignancy, or a prior history of bleeding, are known to be high bleeding risk factors among patients undergoing PCI and receiving DAPT [9]. However, the re-bleeding rate and its risk factors in patients with a history of GIB during DAPT are rarely known. Moreover, when the bleeding focus was limited to the UGI tract, data regarding re-bleeding from NVUGIB have not yet been reported.

Therefore, in this study, we investigated the re-bleeding rate and risk factors of NVUGIB in patients with a history of NVUBIG during DAPT. We also determined the all-cause and re-bleeding-related mortality rates in this population group.

## 2. Materials and Methods

### 2.1. Patients

We retrospectively enrolled patients who were receiving DAPT as a regular medication and were admitted for the treatment of NVUGIB between 2006 and 2020 in a tertiary hospital. All patients received DAPT with confirmed NVUGIB; one was aspirin, and the other antiplatelet agents were clopidogrel, cilostazole, or sarpogrelate (Table 1). Patients taking concurrent anticoagulants such as warfarin or direct oral anticoagulants (DOAC) were excluded. Medication use was evaluated by reviewing the medical records and drug charts. All enrolled patients underwent UGI endoscopy due to (1) symptoms of UGI bleeding, such as hematemesis, melena, or black tarry stool, or (2) a decrease in serum hemoglobin (Hb) greater than 2 g/dL from baseline. NVUGIB was defined as (1) current active bleeding or (2) fresh bloody material with a definite bleeding focus in the UGI tract. All index NVUGIB events were stabilized using endoscopic or conservative management. Patients who had symptoms, UGI bleeding, or significant anemia but did not undergo UGI endoscopy or who were confirmed to have bleeding from the lower GI tract by colonoscopy were excluded.

### 2.2. Parameters

We collected data regarding major comorbidities (heart failure, ischemic heart disease, diabetes, chronic kidney disease [CKD], liver cirrhosis, malignancy, cerebrovascular attack, and hypertension), the Charlson comorbidity index, which is a validated and highly predictive index for mortality [10], a previous history of UGI bleeding, a combination of other drugs such as nonsteroidal anti-inflammatory drugs (NSAIDs) or steroids, location and cause of bleeding, vital signs (heart rate and blood pressure), laboratory findings (Hb, platelet count, international normalized ratio [INR]), renal function, scoring systems of UGI bleeding (Rockall score [RS], Glasgow–Blatchford score [GBS]), treatment modalities, status of *Helicobacter pylori* (*H. pylori*), the length of hospital stay and resumption of antiplatelet agents. The Charlson comorbidity score, RS and GBS were calculated according to previously described formula (Table 1) [10,11,12]. This study was approved by the Institutional Review Board of Korea University, Guro Hospital (IRB number: 2022GR0318).

### 2.3. Study Outcomes

We investigated recurrent NVUGIB and all-cause and re-bleeding-related mortality in the enrolled patients. Clinically significant re-bleeding was defined as the identical criteria of index NVUGIB after 24 h of clinical stability, which was if (1) the patients had symptoms of UGI bleeding or a decrease in serum Hb of ≥2 g/dL from the previous level, and (2) endoscopy showed a current active bleeding or fresh bloody materials with a definite bleeding focus in UGI tract. The total number of re-bleeding episodes per patient was also counted.

### 2.4. Statistical Analysis

Continuous data were assessed using Student’s *t*-test or the Mann–Whitney U test and discontinuous data were assessed using the chi-square test. Logistic rank analysis was used for multivariate analysis. The re-bleeding rate and all-cause and bleeding-related mortality rates were assessed using Kaplan–Meier survival analysis and the log-rank test. A *p*-value less than 0.05 was considered statistically significant. SPSS 22.0 (SPSS Inc., Chicago, IL, USA) was used for statistical analysis.

## 3. Results

### 3.1. Patients and Baseline Characteristics

A total of 124 patients were hospitalized due to NVUGIB during DAPT and were enrolled in this study (mean age: 69.4 years, male: 103, 83.1%, female: 21, 16.9%). Among them, 111 patients (89.5%) took aspirin with clopidogrel, and 13 took aspirin with other antiplatelet agents such as cilostazol and sarpogrelate. More than half of the patients had hypertension (84, 67.7%) and ischemic heart disease (68, 54.8%), and the mean Charlson comorbidity index was 5.4 ± 2.6. Eight patients (6.5%) had a history of UGI bleeding and 16 were taking other drugs that may cause UGI injury, such as NSAIDs or steroids.

After successful hemostasis of the index NVUGIB, we analyzed the clinical course of the enrolled patients. A total of 62 re-bleeding episodes occurred during the 40.7 ± 40.8 months of follow-up, and 36 patients had at least one episode of re-bleeding (29.0%). The mean number of bleeding episodes per patient was 1.7 ± 1.3, and the mean duration from index bleeding to re-bleeding was 12.9 ± 18.0 months. Among them, 12 patients showed early re-bleeding within 30 days from index bleeding, and 24 patients corresponded to late re-bleeding (beyond 30 days from index bleeding). Compared with the non-re-bleeding group, patients in the re-bleeding group were significantly older (73.3 vs. 67.8, *p* = 0.010) and CKD was more frequent (11/36 vs. 13/88, *p* = 0.043) than those in the non-re-bleeding group. The Charlson comorbidity score was marginally higher in re-bleeding group than non-re-bleeding group (6.0 vs. 5.1, *p* = 0.063). However, other variables, such as sex, type of DAPT, other major comorbidities such as heart failure, ischemic heart disease, diabetes, liver cirrhosis, underlying malignancy, cerebrovascular accident, hypertension, symptoms, previous UGI bleeding, and a combination of NSAID or steroids were not significantly different between the two groups (Table 2).

### 3.2. Clinical Characteristics and Outcomes

Most index NVUGIB cases occurred in the stomach, followed by the duodenum and esophagus, and peptic ulcers. Endoscopic hemostasis was performed in 44 patients (35.5%), and RBC transfusion was performed in 86 patients (69.9%). The mean length of hospital stay was 8.9 ± 11.5 days, and most patients (108/115, 93.9%) restarted antiplatelet agents after clinical stabilization. Compared with the non-re-bleeding group, patients in the re-bleeding group showed a lower serum Hb level (7.8 vs. 9.0, *p* = 0.009); however, other clinical characteristics such as the location and cause of bleeding, vital signs, platelet count, international normalized ratio, renal function, RS and GBS, frequency of endoscopic or angiography hemostasis, transfusion, *Helicobacter pylori* positivity, length of hospital stay and resumption of antiplatelet agents were not significantly different between the two groups (Table 3). Multivariate analysis showed that age was the only significant risk factor for re-bleeding (odds ratio [OR], 1.050; 95% confidence interval [CI]: 1.001–1.102, *p* = 0.047) (Table 4).

Nineteen of the 124 patients (15.3%) died during the follow-up period, and re-bleeding was the main cause of death in seven patients (36.8%). Univariate analysis showed that age (76.2 vs. 68.1, *p* = 0.001), underlying malignancy (63.2 vs. 31.4%, *p* = 0.008), and rebleeding episode (47.4 vs. 25.7%, *p* = 0.056) were significantly or marginally different between the dead and undead patients (Supplementary Table S1). Multivariate analysis showed that age (OR, 1.096; 95% CI: 1.020–1.178, *p* = 0.013) and underlying malignancy (OR, 3.646; 95% CI: 1.103–12.053, *p* = 0.024) were significant risk factors for all-cause mortality (Table 5). Similarly, when comparing the re-bleeding-related dead and undead patients, age (79.6 vs. 68.7, *p* = 0.013), underlying malignancy (71.4 vs. 31.4%, *p* = 0.030), and re-bleeding episodes (71.4 vs. 25.7, *p* = 0.010) were significantly different between the two groups (Supplementary Table S2). However, multivariate analysis showed that only age was a significant risk factor for re-bleeding-related mortality (OR, 1.187; 95% CI: 1.032–1.364, *p* = 0.016) (Table 6).

**Figure 1:**
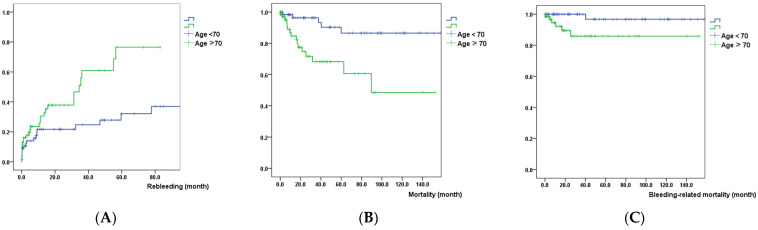
Kaplan–Meier graph that analyzed the differences in (**A**) re-bleeding, (**B**) mortality, and (**C**) re-bleeding related mortality between patients who were aged >70 years and those with age ≤70 years.

Kaplan–Meier analysis showed that patients with an age > 70 years had a significantly higher probability of re-bleeding, mortality, and re-bleeding-related mortality, compared with those with an age ≤ 70 years (*p* = 0.008, <0.001, 0.009, respectively) (Figure 1).

## 4. Discussion

We found that 29.0% of the patients with a history of NVUGIB during DAPT had at least one episode of re-bleeding. Among the 19 patients who died during the follow-up period after stabilization of the index NVUGIB event, 7 (36.8%) had re-bleeding-related mortality. To the best of our knowledge, this is the first study to report the re-bleeding rate in patients with a history of NVUBIG during DAPT. The strength of our study is that all NVUBIG events were confirmed using endoscopic examination and did not rely solely on laboratory findings such as anemia or patient symptoms such as melena or hematemesis.

Aspirin has antiplatelet activity through the inhibition of the cyclooxygenase (COX) enzyme and inhibits COX-1 more than COX-2 by 8–10 times, which can cause a reduction in mucosal blood flow, the inhibition of production of mucus or bicarbonate, and the inhibition of platelet aggregation, and eventually leads to mucosal injury and hemorrhage of the UGI tract [13]. Furthermore, the combination of aspirin with other antiplatelet agents has a synergistic effect on hemorrhage from the UGI [7,14,15]. Thus, physicians who prescribe DAPT should be cautious about the occurrence of UGI bleeding and its prognosis, focusing not only on cardiovascular events but also on recurrent GI bleeding. However, previous studies have not showed the long-term clinical course after the index NVUGIB by DAPT [14], or have only focused on post-NVUGIB cardiovascular events, and have not demonstrated data regarding recurrent UGI bleeding [7,15].

Several scoring systems have been developed for patients with NVUBIG, including the Rockall score (RS), Glasgow–Blatchford score (GBS), and AIMS65 score, which have been validated as useful for predicting the risk of re-bleeding or mortality [12,16]. However, our study did not demonstrate the significance of RS or GBS in predicting re-bleeding, mortality, or re-bleeding-related mortality. As no study has shown the effectiveness of RS or GBS among patients with NVUGIB treated with DAPT, it is unclear whether both systems are useful for this subpopulation. One possibility is that our study enrolled a small number of patients (124) to reach statistical significance. Considering that age is included as a factor for RS, a small number of patients may contribute to the underestimation of the significance of previously established scoring systems. Our data showed that GBS was higher in the re-bleeding group (10.7 vs. 9.3, *p* = 0.119) and RS and GBS were higher in the bleeding-related mortality group (6.9 vs. 5.9, *p* = 0.203; 10.7 vs. 9.9, *p* = 0.543) even though they failed to reach statistical significance, which suggests the hypothesis that RS and GBS would be good predictive tools for re-bleeding and bleeding-related mortality in NVUGIB patients while taking DAPT if a sufficient number of patients were enrolled. Another possibility is that other factors except age that compose RS or GBS, such as vital sign, comorbidities except CKD, the cause of bleeding, endoscopic findings, laboratory findings except hemoglobin, and the presence of melena, were not significantly different between two groups.

Our study showed that only age was a significant risk factor for re-bleeding, all-cause, and re-bleeding-related mortality. Kaplan–Meier graphs showed that patients aged >70 years had a significant probability of re-bleeding, death, and re-bleeding-related death compared to patients aged <70 years. These findings suggest that age is a simple and reliable prognostic factor in patients with NVUGIB during DAPT. Previous studies have reported that age is a significant risk factor for GI hemorrhage and all-cause or cardiac mortality in patients receiving DAPT [7,14,15,17,18]. Physicians need to be alert to re-bleeding and the associated mortality if an elderly patient taking DAPT is hospitalized for NVUGIB. There are several possible reasons for this finding. For example, elderly patients tend to less express alarming symptoms suggesting acute upper GI bleeding, such as abdominal pain or epigastric soreness. Moreover, they frequently have major comorbidities such as CKD, hypertension, diabetes, heart diseases or cerebrovascular diseases. These factors may be combined with age and contribute to re-bleeding and associated mortality in NVUGIB patients who were taking DAPT. It was also interesting that re-bleeding episodes were marginally associated with re-bleeding-related mortality, even though it did not reach statistical significance (OR, 6.161, 95%; CI: 0.957–39.660, *p* = 0.056), which might have been caused by the small sample size. Recurrent bleeding episodes may be a sign of associated mortality, and physicians should be aware of this.

To reduce the UGI complications associated with DAPT, the concomitant use of acid inhibitors such as proton pump inhibitors (PPIs) is recommended [15]. However, it is unclear whether this strategy is effective in preventing re-bleeding in patients with NVUGIB treated with DAPT. In our study, most patients were administered concomitant acid inhibitors, including PPIs, after the onset of the index NVUGIB; however, we could not find any significance of the post-NVUGIB use of acid inhibitors for the prevention of recurrent bleeding. A nationwide Japanese study showed a 6-month incidence of UGI bleeding (3.1–4.2%) among patients who were taking multiple antithrombotic agents, and vonoprazan, a potassium-competitive acid blocker (P-CAB), which is a more potent acid inhibitor, was non-inferior to conventional PPIs for the prevention of 6-month UGI bleeding [19]. Recently, several other P-CABs have shown clinical efficacy in the management of gastroesophageal reflux disorders [20,21] and peptic ulcer disease [22]. P-CABs administered during DAPT are expected to show non-inferior or even superior effectiveness in preventing index or recurrent NVUGIB. Further studies are required to elucidate this issue in the near future.

This study had several limitations. First, this was a retrospective single-center study with a relatively small sample size. Thus, the significance of several factors, such as RS, GBS, CKD, underlying malignancy, or the Charlson comorbidity score, could have been underestimated compared with the real clinical importance. Second, the impairment of other systemic functions after the NVUGIB index, such as cardiac, renal, or pulmonary function, was not estimated, which could also significantly affect the prognosis of the patients. However, we found that more than one-third of the mortality was associated with recurrent bleeding, even after clinical stabilization of the NVUGIB index during DAPT. Third, most of the enrolled patients were taking the combination of aspirin plus clopidogrel (111, 89.5%) and a relatively small number of patients (13, 10.5%) were taking aspirin plus other P2Y12 inhibitors such as cilostazol or sarpogrelate, which are more potent than clopidogrel. Moreover, we did not include patients who were taking newly developed and more potent P2Y12 inhibitors such as ticagrelol and prasugrel, which may cause more severe bleeding complications than clopidogrel.

## 5. Conclusions

Some patients with NVUGIB during DAPT may experience recurrent UGI bleeding, and age is a simple and reliable factor for the prediction of re-bleeding and all-cause and bleeding-related deaths during follow-up. Further large-scale prospective studies are expected to show the prognosis of patients with NVUGIB taking DAPT, especially focusing on recurrent GI complications and related mortality.

## Figures and Tables

**Table 1:** Summary of scoring systems for Charlson comorbidity score, Rockall score and Glasgow–Blatchford Score.

Scoring System	Clinical Factor	Parameter	Score
Charlson comorbidity score	Age (year)	50–59	1
		60–69	2
		70–79	3
		≥80	4
	Comorbidity	Myocardial infarction	1
		CHF	1
		Peripheral vascular disease	1
		CVA or TIA	1
		Dementia	1
		COPD	1
		Connective tissue disease	1
		Peptic ulcer disease	1
		Liver disease (mild)	1
		Liver disease (moderate to severe)	3
		Diabetes (uncomplicated)	1
		Diabetes (end-organ disease)	2
		Hemiplegia	2
		Moderate to severe CKD	2
		Solid tumor (localized)	2
		Solid tumor (metastatic)	6
		Leukemia	2
		Lymphoma	2
		AIDS	6
Rockall score	Age (year)	<60	0
		60–79	1
		≥80	2
	Shock	Heart rate >100 bpm	1
		SBP <100 mm Hg	2
	Comorbidity	No major comorbidity	0
		Any comorbidity except renal failure, liver failure, and/or disseminated malignancy	2
		Renal failure, liver failure, and/or disseminated malignancy	3
	Endoscopic finding	Mallory–Weiss tear or no lesion identified and no stigmata of recent hemorrhage	0
		All other diagnoses	1
		Malignancy of upper GI tract	2
	Stigmata of recent bleeding	No stigmata or dark spot only	0
		Blood in upper GI tract, adherent clot, visible or spurting vessel	2
Glasgow–Blatchford score	BUN, mg/dL	≥18.2 to <22.4	2
		≥22.4 to <28	3
		≥28 to <70	4
		≥70	6
	Hemoglobin level, g/dL	Male ≥12.0 to <13.0	1
		≥10.0 to <12.0	3
		<10.0	6
		Female ≥10.0 to <12.0	1
		<10.0	6
	SBP, mm Hg	≥100 to <109	1
		≥90 to <100	2
		<90	3
	Other markers	Heart rate >100 bpm	1
		Presented with melena	1
		Presented with syncope	2
		Hepatic disease	2
		Cardiac failure	2

CHF, congestive heart failure; CVA, cerebrovascular accident; TIA, transient ischemic attack; COPD, chronic obstructive pulmonary disease; CKD, chronic kidney disease; AIDS, acquired immunodeficiency syndrome; SBP, systolic blood pressure; GI, gastrointestinal.

**Table 2:** Baseline characteristics.

	Re-Bleeding (−) (*n* = 88)	Re-Bleeding (+) (*n* = 36)	Total (*n* = 124)	*p*-Value *
Age (year, ±SD)	67.8 ± 11.8	73.3 ± 7.3	69.4 ± 10.9	0.010
Male: female, *n*	74:14	29:7	103:21	0.634
DAPT type, *n* (%)				0.224
Aspirin + clopidogrel	81 (92.0)	30 (83.3)	111 (89.5)	
Aspirin + others **	7 (8.0)	6 (16.7)	13 (10.5)	
Comorbidities, *n* (%)				
Heart failure	11 (12.5)	4 (11.1)	15 (12.1)	0.830
Ischemic heart disease	50 (56.8)	18 (50.0)	68 (54.8)	0.489
Diabetes	32 (36.8)	18 (50.0)	50 (40.3)	0.160
Chronic kidney disease	13 (14.8)	11 (30.6)	24 (19.4)	0.043
Liver cirrhosis	3 (3.4)	1 (2.8)	4 (3.2)	0.857
Underlying malignancy	30 (34.1)	15 (41.7)	45 (36.3)	0.426
Cerebrovascular attack	32 (36.4)	13 (36.1)	45 (36.3)	0.979
Hypertension	58 (65.9)	26 (72.2)	84 (67.7)	0.495
Charlson comorbidity index (± SD)	5.1 ± 2.6	6.0 ± 2.5	5.4 ± 2.6	0.063
Symptom or sign, *n* (%)				0.515
Anemia	24 (27.3)	7 (19.4)	31 (25.0)	
Hematemesis	13 (14.8)	3 (8.3)	16 (12.9)	
Melena	49 (55.7)	25 (69.4)	74 (59.7)	
Hematochezia	2 (2.3)	1 (2.8)	3 (2.4)	
Previous UGI bleeding history, *n* (%)	6 (6.8)	2 (5.6)	8 (6.5)	0.795
Combination of other drug ***, *n* (%)	13 (14.8)	3 (8.9)	16 (12.9)	0.332

SD, standard deviation; DAPT, dual anti-platelet therapy; UGI, upper gastrointestinal. * Comparing rebleeding (−) and (+) groups. ** Includes cilostazole and sarpogrelate. *** Includes nonsteroidal anti-inflammatory drug or steroid.

**Table 3:** Clinical characteristics of initial NVUGIB.

	Re-Bleeding (−) (*n* = 88)	Re-Bleeding (+) (*n* = 36)	Total (*n* = 124)	*p*-Value *
Location of bleeding, *n* (%)				0.573
Esophagus	6 (6.8)	1 (2.8)	7 (5.6)	
Stomach	67 (76.1)	27 (75.0)	94 (75.8)	
Duodenum	15 (17.0)	8 (22.2)	23 (18.5)	
Cause of bleeding, *n* (%)				0.199
Peptic ulcer	51 (58.0)	21 (58.3)	72 (58.1)	0.965
Active bleeding or exposed vessel	27 (52.9)	11 (52.4)	38 (52.8)	
Fresh or old blood clots	24 (47.1)	10 (47.6)	34 (47.2)	
UGI malignancy	26 (29.5)	8 (22.2)	34 (27.4)	
Mallory-Weiss	4 (4.5)	1 (2.8)	5 (4.0)	
Angiodysplasia	5 (5.7)	1 (2.8)	6 (4.8)	
Anastomosis site bleeding	1 (1.1)	3 (8.3)	4 (3.2)	
Others **	1 (1.1)	2 (5.6)	3 (2.4)	
Heart rate (/minute, ±SD)	89.0 ± 17.3	85.6 ± 13.7	88.2 ± 16.4	0.247
SBP (mmHg, ± SD)	118.2 ± 22.8	112.2 ± 21.6	116.5 ± 22.5	0.171
Hemoglobin (g/dL, ±SD)	9.0 ± 2.3	7.8 ± 2.1	8.6 ± 2.3	0.009
Platelet count (×10^3^/µL, ±SD)	257.5 ± 132.1	229.5 ± 81.3	249.4 ± 120.0	0.240
International normalized ratio	1.1 ± 0.1	1.1 ± 0.2	1.1 ± 0.1	0.668
BUN (mg/dL, ±SD)	34.0 ± 23.0	42.1 ± 29.0	36.3 ± 24.9	0.100
Serum creatinine (mg/dL, ±SD)	1.5 ± 2.0	1.5 ± 1.2	1.5 ± 1.8	0.904
eGFR (mL/min/1.73 m^2^, ±SD)	84.2 ± 40.1	72.6 ± 40.7	80.8 ± 40.4	0.152
Rockall score	5.9 ± 1.6	6.0 ± 1.5	6.0 ± 1.6	0.337
Glasgow–Blatchford score	9.6 ± 3.6	10.7 ± 3.4	9.9 ± 3.6	0.116
Endoscopic findings, *n* (%)				0.949
Active bleeding or exposed vessel	41 (46.6)	17 (47.2)	58 (46.8)	
Fresh or old blood clots	47 (53.4)	19 (52.8)	66 (53.2)	
Endoscopic hemostasis, *n* (%)	29 (33.0)	15 (41.7)	44 (35.5)	0.357
Angiographic hemostasis, *n* (%)	2 (2.3)	1 (2.8)	3 (2.4)	0.868
RBC transfusion, *n* (%)	57 (65.5)	29 (80.6)	86 (69.9)	0.098
*H. pylori* positivity, *n* (%)	23/53 (43.4)	8/24 (33.3)	31/77 (40.3)	0.404
Length of hospital stay (days, ±SD)	7.9 ± 9.7	11.1 ± 14.4	8.9 ± 11.5	0.130
Resumption of antiplatelet agent, *n* (%)	78/81 (96.3)	30/34 (88.2)	108/115 (93.9)	0.099

NVUGIB, non-variceal upper gastrointestinal bleeding; UGI, upper gastrointestinal; SD, standard deviation; SBP, systolic blood pressure; BUN, blood urea nitrogen; eGFR, estimated glomerular filtration rate; RBC, red blood cell; *H. pylori*, *Helicobacter pylori.* * Comparing rebleeding (−) and (+) groups. ** Includes erosion and post-endoscopic sphincterotomy bleeding.

**Table 4:** Multivariate regression analysis for rebleeding.

Variables		95% Confidence Interval		
	Odd Ratio	Upper Limit	Lower Limit	*p*-Value
Age	1.050	1.001	1.102	0.047
Chronic kidney disease	1.667	0.558	4.981	0.360
Charlson comorbidity score	1.028	0.849	1.244	0.777
Hemoglobin level	0.831	0.675	1.023	0.081

Note: adjusted factors included age, chronic kidney disease, Charlson comorbidity score and hemoglobin level.

**Table 5:** Multivariate regression analysis for all-cause mortality.

Variables		95% Confidence Interval		
	Odd Ratio	Upper Limit	Lower Limit	*p*-Value
Age	1.096	1.020	1.178	0.013
Underlying malignancy	3.646	1.103	12.053	0.024
Charlson comorbidity score	1.047	0.823	1.331	0.711
Rebleeding episode	2.028	0.687	5.981	0.200

Note: adjusted factors included age, underlying malignancy, Charlson comorbidity score and rebleeding episode.

**Table 6:** Multivariate regression analysis for rebleeding-related mortality.

Variables		95% Confidence Interval		
	Odd Ratio	Upper Limit	Lower Limit	*p*-Value
Age	1.187	1.032	1.364	0.016
Underlying malignancy	8.316	0.900	76.882	0.062
Charlson comorbidity score	0.978	0.664	1.440	0.909
Rebleeding episode	5.585	0.837	37.284	0.076

Note: adjusted factors included age, underlying malignancy, Charlson comorbidity score and rebleeding episode.

## Data Availability

The data are not publicly available because of our institutional guidelines. The data presented in this study are available on request from the corresponding author.

## Supplementary Materials

The following supporting information can be downloaded at: https://www.mdpi.com/article/10.3390/diagnostics13223444/s1, Supplementary Table S1. Baseline and clinical characteristics according to all-cause mortality, Supplementary Table S2. Baseline and clinical characteristics according to rebleeding-related mortality.
