# Supplementary material for: Recurrent Non-Variceal Upper Gastrointestinal Bleeding among Patients Receiving Dual Antiplatelet Therapy
Source: Diagnostics (Basel). 2023 Nov 14;13(22):3444. doi: 10.3390/diagnostics13223444 (PMC10670490; doi:10.3390/diagnostics13223444)
Supplement: Supplementary file 1 [file diagnostics-13-03444-s001.zip › diagnostics-2608013-supplementary.pdf]

Supplementary Table S1. Baseline and Clinical Characteristics according to All-Cause Mortality

	Mortality (-) (n = 105)	Mortality (+) (n = 19)	Total (n = 124)	*P*-value[*]
Age (year, ± SD)	68.1 ± 11.0	76.2 ± 8.1	69.4 ± 10.9	0.001
Male: female, n	89:16	14:5	103:21	0.236
DAPT type, n (%)				1.000
Aspirin + clopidogrel	94 (89.5)	17 (89.5)	111 (89.5)	
Aspirin + others[**]	11 (10.5)	2 (10.5)	13 (10.5)	
Comorbidities, n (%)				
Heart failure	12 (11.4)	3 (15.8)	15 (12.1)	0.592
Ischemic heart disease	59 (56.2)	9 (47.4)	68 (54.8)	0.477
Diabetes	41 (39.0)	9 (47.4)	50 (40.3)	0.496
Chronic kidney disease	20 (19.0)	4 (21.1)	24 (19.4)	0.839
Liver cirrhosis	4 (3.8)	0 (0.0)	4 (3.2)	0.387
Underlying malignancy	33 (31.4)	12 (63.2)	45 (36.3)	0.008
Cerebrovascular attack	37 (35.2)	8 (42.1)	45 (36.3)	0.567
Hypertension	72 (68.6)	12 (63.2)	84 (67.7)	0.624
Charlson comorbidity index (± SD)	5.1 ± 2.6	6.0 ± 2.5	5.4 ± 2.6	0.063
Symptom or sign, n (%)				0.098
Anemia	22 (21.0)	9 (47.4)	31 (25.0)	
Hematemesis	14 (13.3)	2 (10.5)	16 (12.9)	
Melena	66 (62.8)	8 (42.1)	74 (59.7)	
Hematochezia	3 (2.9)	0 (0.0)	3 (2.4)	
Previous UGI bleeding history, n (%)	7 (6.7)	1 (5.3)	8 (6.5)	0.819
Combination of other drug[***], n (%)	11 (10.5)	5 (26.3)	16 (12.9)	0.071
Location of bleeding, n (%)				0.457
Esophagus	7 (6.7)	0 (0.0)	7 (5.6)	
Stomach	78 (74.5)	16 (84.2)	94 (75.8)	
Duodenum	20 (19.0)	3 (15.8)	23 (18.5)	
Cause of bleeding, n (%)				0.109
Peptic ulcer	64 (61.0)	8 (42.1)	72 (58.1)	
UGI malignancy	24 (22.9)	10 (52.6)	34 (27.4)	
Mallory-Weiss	5 (4.8)	0 (0.0)	5 (4.0)	
Angiodysplasia	6 (5.7)	0 (0.0)	6 (4.8)	
Anastomosis site bleeding	3 (2.9)	1 (5.3)	4 (3.2)	
Others[**]	3 (2.9)	0 (0.0)	3 (2.4)	
Heart rate (/minute, ± SD)	87.5 ± 17.0	90.7 ± 11.9	88.2 ± 16.4	0.331
SBP (mmHg, ± SD)	117.3 ± 21.3	111.7 ± 24.0	116.5 ± 22.5	0.315
Hemoglobin (g/dL, ± SD)	8.6 ± 2.5	8.9 ± 1.1	8.6 ± 2.3	0.601

Platelet count (×10^3/μL, ± SD)	246.0 ± 119.6	268.4 ± 123.3	249.4 ± 120.0	0.471
International normalized ratio	1.1 ± 0.1	1.1 ± 0.1	1.1 ± 0.1	0.471
BUN (mg/dL, ± SD)	36.8 ± 25.3	33.8 ± 23.6	36.3 ± 24.9	0.634
Serum creatinine (mg/dL, ± SD)	1.5 ± 1.9	1.4 ± 1.2	1.5 ± 1.8	0.875
eGFR (mL/min/1.73 m^2, ± SD)	82.2 ± 40.4	73.3 ± 41.1	80.8 ± 40.4	0.391
Rockall score	5.9 ± 1.5	6.4 ± 1.6	6.0 ± 1.6	0.237
Glasgow-Blatchford score	9.9 ± 3.7	10.3 ± 2.6	9.9 ± 3.6	0.602
Endoscopic findings, n (%)				0.149
Active bleeding or exposed vessel	52 (49.5)	6 (31.6)	58 (46.8)	
Fresh or old blood clots	53 (50.5)	13 (68.4)	66 (53.2)	
Endoscopic hemostasis, n (%)	39 (37.1)	5 (26.3)	44 (35.5)	0.364
Angiographic hemostasis, n (%)	3 (2.9)	0 (0.0)	3 (2.4)	0.456
RBC transfusion, n (%)	74 (71.2)	12 (63.2)	86 (69.9)	0.485
H. pylori positivity, n (%)	30/69 (43.5)	1/8 (12.5)	31/77 (40.3)	0.091
Length of hospital stay (days, ± SD)	8.7 ± 10.7	9.2 ± 15.6	8.9 ± 11.5	0.903
Resumption of antiplatelet agent, n (%)	91/96 (94.8)	17/19 (89.5)	108/115(93.9)	0.376
Rebleeding episode, n (%)	27 (25.7)	9 (47.4)	36 (29.0)	0.056

SD, standard deviation; DAPT, dual anti-platelet therapy; UGI, upper gastrointestinal; SBP, systolic blood pressure; BUN, blood urea nitrogen; eGFR, estimated glomerular filtration rate; RBC, red blood cell; *H. pylori*, *Helicobacter pylori*;

*Comparing rebleeding (-) and (+) groups

**Includes cilostazole and sarpogrelate

***Includes nonsteroidal anti-inflammatory drug or steroid

Supplementary Table S2. Baseline and Clinical Characteristics according to Rebleeding-Related Mortality

	Mortality (-) (n = 105)	Mortality (+) (n = 7)	Total (n = 112)	*P*-value*
Age (year, ± SD)	68.1 ± 11.0	79.6 ± 8.8	68.9 ± 11.1	0.013
Male: female, n	89:16	5:2	94:18	0.352
DAPT type, n (%)				0.966
Aspirin + clopidogrel	94 (89.5)	6 (85.7)	100 (89.3)	
Aspirin + others**	11 (10.5)	1 (14.3)	12 (10.7)	
Comorbidities, n (%)				
Heart failure	12 (11.4)	2 (28.6)	14 (12.5)	0.184
Ischemic heart disease	59 (56.2)	3 (42.9)	62 (55.4)	0.492
Diabetes	41 (39.0)	4 (57.1)	45 (40.2)	0.344
Chronic kidney disease	20 (19.0)	2 (28.6)	22 (19.6)	0.539
Liver cirrhosis	4 (3.8)	0 (0.0)	4 (3.6)	0.599
Underlying malignancy	33 (31.4)	5 (71.4)	38 (33.9)	0.030
Cerebrovascular attack	37 (35.2)	3 (42.9)	40 (35.7)	0.684
Hypertension	72 (68.6)	3 (42.9)	75 (67.0)	0.161
Charlson comorbidity index (± SD)	5.1 ± 2.6	6.0 ± 2.5	5.4 ± 2.6	0.063
Symptom or sign, n (%)				0.154
Anemia	22 (21.0)	4 (57.1)	26 (23.2)	
Hematemesis	14 (13.3)	0 (0.0)	14 (12.5)	
Melena	66 (62.8)	3 (42.9)	69 (61.6)	
Hematochezia	3 (2.9)	0 (0.0)	3 (2.7)	
Previous UGI bleeding history, n (%)	7 (6.7)	0 (0.0)	7 (6.3)	0.480
Combination of other drug***, n (%)	11 (10.5)	1 (14.3)	12 (10.7)	0.752
Location of bleeding, n (%)				0.719
Esophagus	7 (6.7)	0 (0.0)	7 (6.3)	
Stomach	78 (74.5)	6 (85.7)	84 (75.0)	
Duodenum	20 (19.0)	1 (14.3)	21 (18.8)	
Cause of bleeding, n (%)				0.141
Peptic ulcer	64 (61.0)	2 (28.6)	66 (58.9)	
UGI malignancy	24 (22.9)	5 (71.4)	29 (25.9)	
Mallory-Weiss	5 (4.8)	0 (0.0)	5 (4.5)	
Angiodysplasia	6 (5.7)	0 (0.0)	6 (5.4)	
Anastomosis site bleeding	3 (2.9)	0 (0.0)	3 (2.7)	
Others**	3 (2.9)	0 (0.0)	3 (2.7)	
Heart rate (/minute, ± SD)	87.5 ± 17.0	88.4 ± 12.8	87.6 ± 16.8	0.866
SBP (mmHg, ± SD)	117.3 ± 21.3	110.9 ± 28.1	117.0 ± 22.6	0.569
Hemoglobin (g/dL, ± SD)	8.6 ± 2.5	8.8 ± 0.9	8.6 ± 2.4	0.835

Platelet count (×10³/μL, ± SD)	246.0 ± 119.6	276.6 ± 149.4	247.9 ± 121.1	0.613
International normalized ratio	1.1 ± 0.1	1.1 ± 0.2	1.1 ± 0.1	0.416
BUN (mg/dL, ± SD)	36.8 ± 25.3	30.3 ± 16.2	36.4 ± 24.8	0.368
Serum creatinine (mg/dL, ± SD)	1.5 ± 1.9	1.5 ± 1.4	1.5 ± 1.9	0.915
eGFR (mL/min/1.73 m², ± SD)	82.2 ± 40.4	66.7 ± 42.6	81.2 ± 40.5	0.385
Rockall score	5.9 ± 1.5	6.9 ± 1.8	6.0 ± 1.6	0.203
Glasgow-Blatchford score	9.9 ± 3.7	10.7 ± 1.3	9.9 ± 3.6	0.543
Endoscopic findings, n (%)				0.733
Active bleeding or exposed vessel	52 (49.5)	3 (42.9)	55 (49.1)	
Fresh or old blood clots	53 (50.5)	4 (57.1)	57 (50.9)	
Endoscopic hemostasis, n (%)	39 (37.1)	2 (28.6)	41 (36.6)	0.649
Angiographic hemostasis, n (%)	3 (2.9)	0 (0.0)	3 (2.7)	0.650
RBC transfusion, n (%)	74 (71.2)	5 (71.4)	79 (71.2)	0.988
H. pylori positivity, n (%)	30/69 (43.5)	0/2 (0.0)	30/71 (42.3)	0.220
Length of hospital stay (days, ± SD)	8.7 ± 10.7	5.0 ± 3.9	8.4 ± 10.4	0.063
Resumption of antiplatelet agent, n (%)	91/96 (94.8)	5/7 (71.4)	96/103 (93.2)	0.071
Rebleeding episode, n (%)	27 (25.7)	5 (71.4)	32 (28.6)	0.010

SD, standard deviation; DAPT, dual anti-platelet therapy; UGI, upper gastrointestinal; SBP, systolic blood pressure; BUN, blood urea nitrogen; eGFR, estimated glomerular filtration rate; RBC, red blood cell; *H. pylori*, *Helicobacter pylori*;

*Comparing rebleeding (-) and (+) groups

**Includes cilostazole and sarpogrelate

***Includes nonsteroidal anti-inflammatory drug or steroid
